# Intermediates in the Sox sulfur oxidation pathway are bound to a sulfane conjugate of the carrier protein SoxYZ

**DOI:** 10.1371/journal.pone.0173395

**Published:** 2017-03-03

**Authors:** Daniel B. Grabarczyk, Ben C. Berks

**Affiliations:** Department of Biochemistry, University of Oxford, Oxford, United Kingdom; Martin-Luther-Universitat Halle-Wittenberg, GERMANY

## Abstract

The Sox pathway found in many sulfur bacteria oxidizes thiosulfate to sulfate. Pathway intermediates are covalently bound to a cysteine residue in the carrier protein SoxYZ. We have used biochemical complementation by SoxYZ-conjugates to probe the identity of the intermediates in the Sox pathway. We find that unconjugated SoxYZ and SoxYZ-S-sulfonate are unlikely to be intermediates during normal turnover in disagreement with current models. By contrast, conjugates with multiple sulfane atoms are readily metabolised by the Sox pathway. The most parsimonious interpretation of these data is that the true carrier species in the Sox pathway is a SoxYZ-S-sulfane adduct.

## Introduction

The oxidation of inorganic sulfur compounds by bacteria is a major part of the biogeochemical sulfur cycle. This metabolic process is exploited industrially in the bio-mining of metals [1] and in cleaning fossil fuel emissions [2]. Oxidation of sulfur species provides the bacteria with reductant for use either in carbon dioxide fixation or for energy conservation through membrane-bound electron transport chains [3, 4].

**Fig 1:**
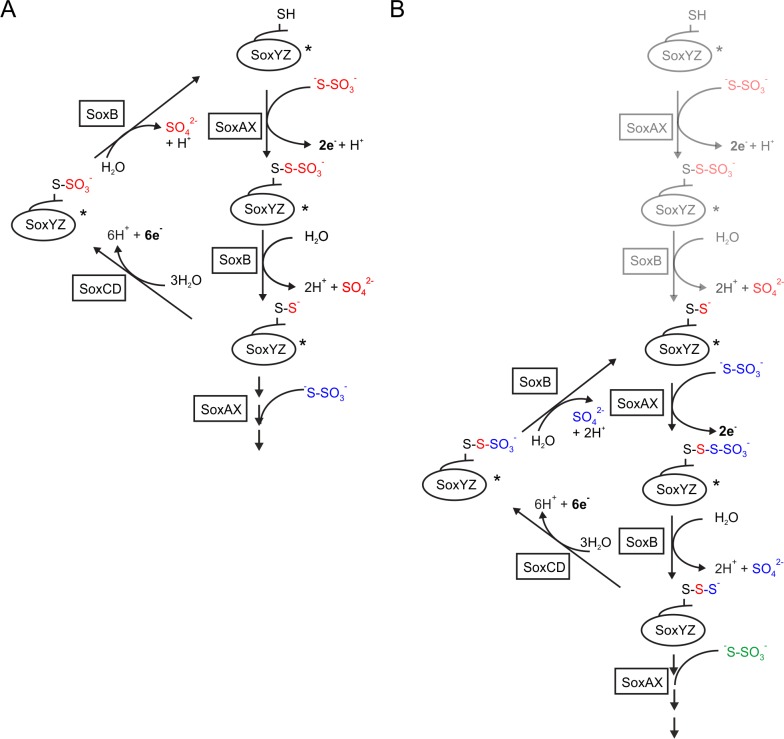
Models for the Sox pathway. (A) The current Sox pathway model. The pathway cycle starts with an unmodified SoxYZ Cys side chain (top of the panel). Released electrons are transferred to cytochrome *c*. SoxYZ conjugates with longer chain sulfur species are generated by competition between SoxAX and SoxCD for SoxY(S)Z (bottom of the panel). SoxYZ species that have been identified in extracts of thiosulfate-grown *P*. *pantotrophus* [5] are marked with an asterisk (*). (B) A revised model for the Sox pathway. The canonical Sox pathway model shown in (A) is modified to accommodate the results of the current work. The reactions in gray allow the activation of unconjugated SoxYZ.

The Sox pathway found in many sulfur-oxidizing bacteria oxidizes the sulfur-containing molecule thiosulfate. Intermediates in the Sox pathway are covalently attached to the heterodimeric carrier protein SoxYZ through a cysteine in a swinging arm on the SoxY protein [5–7]. In the canonical Sox pathway of *Paracoccus pantrophus* thiosulfate is completely oxidised to sulfate with the eight electrons released being transferred to a small *c*-type cytochrome for input to the electron transport chain. Work from a number of groups has led to the development of a consensus model for the *P*. *pantotrophus* Sox pathway (Fig 1A) [3]. In this model thiosulfate is initially oxidatively conjugated to SoxYZ by the enzyme SoxAX to produce SoxYZ-S-thiosulfonate [8, 9]. Next, the terminal sulfonate group is hydrolytically removed by the enzyme SoxB [7, 10, 11] with the newly-exposed terminal sulfane group then oxidized to the corresponding sulfonate by the enzyme SoxCD [12, 13]. Finally, the sulfonate group is removed by the action of SoxB.

The Sox pathway model was developed on the basis of three types of observation. Firstly, that it is possible to reconstitute the complete eight electron oxidation of thiosulfate by mixing the purified Sox components together [14]. Thus, the known Sox components are necessary and sufficient to catalyse the full Sox pathway reaction. The electron yield of the pathway falls to two if SoxCD is omitted [14], as predicted by the pathway model. Secondly, the proposed pathway intermediates SoxYZ-S-sulfane (‘SoxY(S)Z’), SoxYZ-S-sulfonate (‘SoxY(SO_3_)Z’), SoxYZ-S-thiosulfonate (‘SoxY(SSO_3_)Z’), as well as SoxYZ-S-thioperoxosulfonate (‘SoxY(SSSO_3_)Z’), can all be identified in SoxYZ preparations isolated from cells metabolising thiosulfate [5]. Thirdly, each Sox pathway enzyme is homologous to an enzyme of known function and has been assigned a related catalytic reaction in the Sox pathway model. SoxAX is related to the thiosulfate dehydrogenase TsdA, which catalyses the oxidative conjugation of two thiosulfate molecules through formation of a disulfide linkage [15, 16]. SoxB is a member of a family of hydrolytic enzymes containing a divalent metal ion pair at the active site [17]. SoxCD is related to sulfite oxidases [12]. Consistent with these inferred catalytic similarities SoxB has been shown to have thiosulfohydrolase activity using the small-molecule substrate analogue trithionate [7] while SoxCD is able to catalyse sulfite oxidation [13]. Nevertheless, it is important to realise that none of the proposed reactions of the Sox pathway has been directly demonstrated. Indeed, the individual purified Sox enzymes do not turn over their predicted SoxYZ-conjugated substrates ([7, 18] and observations detailed below).

In this work we have probed the identity of the intermediates in the Sox pathway by characterizing the ability of the reconstituted *P*. *pantotrophus* Sox system to metabolise defined SoxYZ-conjugates. This approach shows that SoxYZ-S-sulfonate is not a pathway intermediate and that unconjugated SoxYZ is unlikely to be an intermediate of the pathway during normal turnover. By contrast conjugates with multiple sulfane atoms are found to be readily metabolised by the reconstituted Sox system. The most parsimonious interpretation of these data is that a sulfane-modified form of SoxYZ is the normal carrier for the intermediates in the Sox pathway.

## Materials and methods

### Production of SoxYZ conjugates

Recombinant his_6_-tagged *Thermus thermophilus* SoxY(SSO_3_)Z and strep-tagged *T*. *thermophilus* SoxB were produced and purified as described previously [11] using codon-optimized expression plasmids [7]. The sequences of the codon-optimized genes have been deposited with the accession numbers KY421729 (*soxB*), KY421730 (*soxY*), and KY421731 (*soxZ*). The SoxY(SO_3_)Z conjugate was generated by reacting SoxY(SSO_3_)Z with 50 mM sodium sulfite for 30 minutes and then removing small molecules by size exclusion chromatography on a Superdex 75 10/300 column (GE healthcare) in ITC buffer (30 mM Tris/HCl pH 8.0, 200 mM NaCl). The plasmid used to produce the SoxY_C151E_Z variant was constructed using Quikchange mutagenesis (Stratagene) with the primers 5´-GACCGTGGGCGGCGAAGGCTGAGGTACC-3´ and 5´-GGTACCTCAGCCTTCGCCGCCCACGGTC-3´.

Recombinant his_6_-tagged *P*. *pantotrophus* SoxYZ was produced and purified as described previously [6] with the following modifications. 2 mM TCEP (tris[2-carboxyethyl]phosphine) was added to all buffers to the end of the immobilized metal affinity chromatography step. The pooled SoxYZ-containing fractions were then supplemented with 20 mM TCEP and the subsequent size exclusion chromatography step was run with no TCEP in the running buffer. The resulting SoxYZ sample is termed ‘TCEP-treated SoxYZ’.

‘Tetrathionate-treated SoxYZ’ was generated from TCEP-treated *P*. *pantotrophus* SoxYZ by reacting purified SoxYZ with 50 mM potassium tetrathionate for one hour at room temperature then removing small molecules by size exclusion chromatography on a Superdex 75 10/300 column in SEC buffer (30 mM Tris/HCl pH 8.0, 160 mM NaCl). SoxY(SO_3_)Z was generated by reacting tetrathionate-treated SoxYZ with 50 mM sodium sulfite for 30 min followed by size exclusion chromatography on a Superdex 75 10/300 column equilibrated in SEC buffer.

‘Sulfide-treated SoxYZ’ was generated by reacting tetrathionate-treated SoxYZ with 10 mM sodium sulfide for one hour. Excess sodium sulfide was removed using a PD-10 desalting column (GE Healthcare) equilibrated in SEC buffer before further purification of the sample using a Superdex 75 10/300 column equilibrated in SEC buffer.

### Purification of *P*. *pantotrophus* Sox enzymes

*P*. *pantotrophus GB17* was grown under chemolithotrophic conditions with thiosulfate as electron donor. The growth medium contained 0.7 g/l Na_2_HPO_4_, 0.3 g/l KH_2_PO_4_, 1 g/l NH_4_Cl, 0.1 g/l MgSO_4_, 80 mg CaCl_2_, 1 mg ferric citrate, 2 ml/l trace element solution [19], 20 mM sodium thiosulfate, 12 mM sodium bicarbonate, and 1 mg/ml phenol red. 500 mL cultures were grown in 2.5 L flasks at 30°C in a shaking incubator. When the culture pH fell below neutrality, as judged from the phenol red indicator, additional sodium bicarbonate was added to a final concentration of 12 mM. Every second bicarbonate addition was accompanied by the addition of 20 mM sodium thiosulfate. Cultures were allowed to reach an OD_600_ = 0.6 and then harvested by centrifugation. The pellet was resuspended in 55 mM potassium phosphate pH 7.5, supplemented with Roche EDTA-free protease inhibitor mix and a few crystals of DNase I and lysozyme (both Sigma-Aldrich). Cells were lysed by two passages through a French Press (ThermoScientific) at 75 MPa. Cell debris was removed by centrifiguation.

SoxB, SoxAX, and SoxCD were purified from the soluble extract by a modification of a published method [14]. All chromatography steps were carried out at 24°C. Starting with the cell extract from ~10 g wet weight of cells a 44–65% saturation ammonium sulfate fraction was isolated at 4°C, dialysed against 25 mM sodium potassium phosphate pH 6.5 (Buffer A) at 4°C, loaded onto a 1 mL Q-sepharose column (GE Healthcare) pre-equilibrated in Buffer A, and then washed with 10 column volumes of Buffer A. SoxYZ was removed by washing the column with 0.2 M NaCl in Buffer A. A 0.2 M to 1 M NaCl gradient over 60 column volumes in Buffer A was used to elute the other Sox components. The Sox enzymes were identified using SDS-PAGE and staining for protein with Coomassie Blue or for covalently-bound heme by detecting microperoxidase activity [20]. The presence of the enzymes was confirmed by reconstitution assays. Individual fractions containing SoxB, or SoxAX or SoxCD were subject to size-exclusion chromatography using a Superdex 200 10/300 column (GE Healthcare) equilibrated in Buffer A. SoxAX and SoxCD were then further purified by ion-exchange chromatography using a 1 mL MonoQ HR column (GE Healthcare) equilibrated in 25 mM bis-tris pH 6.0. Proteins were eluted with a gradient of 0 to 500 mM NaCl over 50 column volumes. SoxB was brought to 30% saturation ammonium sulfate and loaded on a Hitrap Phenyl HP column (GE Healthcare) equilibrated in Buffer A with 30% saturation ammonium sulfate. SoxB was eluted with a gradient from 30 to 0% saturation ammonium sulfate over 25 column volumes. The purified Sox proteins were concentrated and exchanged into Buffer A by ultrafiltration.

### Electrospray ionization mass spectrometry

Samples for analysis (typically 20μl) were diluted 1:50 in 0.1% (v/v) formic acid and then desalted into 50% v/v acetonitrile/water, 0.1% formic acid using in-line reverse phase chromatography on a Merck Chromolith C18 2 x 5 mm guard column. The samples were then loaded into a Waters LCT Premier electrospray ionisation mass spectrometer (ESI-MS1) operated in positive ion mode. All spectra were acquired and analysed using MassLynx software (Waters)

### Reconstitution of Sox pathway activity

All reconstitution assays were performed at 24°C in 30 mM Tris-HCl pH 8.0, 160 mM NaCl. This buffer resulted in higher activity than the pH 7.5 phosphate buffer previously used by Friedrich and co-workers [14]. Unless otherwise indicated, reactions contained 0.1 μM each of native *P*. *pantotrophus* SoxB, SoxAX and SoxCD, 35 μM horse heart cytochrome *c* (Sigma-Aldrich) and 2 mM sodium thiosulfate. Reactions were initiated by addition of 1 μM recombinant *P*. *pantotrophus* SoxYZ and reduction of cytochrome *c* was followed by an increase in absorption at 550 nm using Δε_550nm_ = 21.1 mM^-1^ cm^-1^.

### Isothermal titration calorimetry

All ITC experiments were performed on a MicroCal iTC_200_ at 25°C with a reference power of 3 cal/s and a stirring speed of 1000 rpm. Experiments were carried out with SoxB in the cell and SoxYZ as the titrant. Both proteins were in ITC Buffer. Control experiments were carried out to confirm that sample dilution did not cause systematic deviation from a flat baseline. Traces were integrated using OriginPro and then two replicate experiments were simultaneously fitted to a hetero-association model (A + B ↔ AB) with ΔH and *K*_D_ as fitting parameters using SEDPHAT [21].

## Results

### Production and characterization of putative Sox pathway intermediates

The reaction scheme shown in Fig 1A predicts that specific SoxYZ-conjugates are intermediates of the Sox pathway. We sought to produce defined preparations of these proposed intermediates in order to experimentally test their involvement in the Sox pathway.

**Fig 2:**
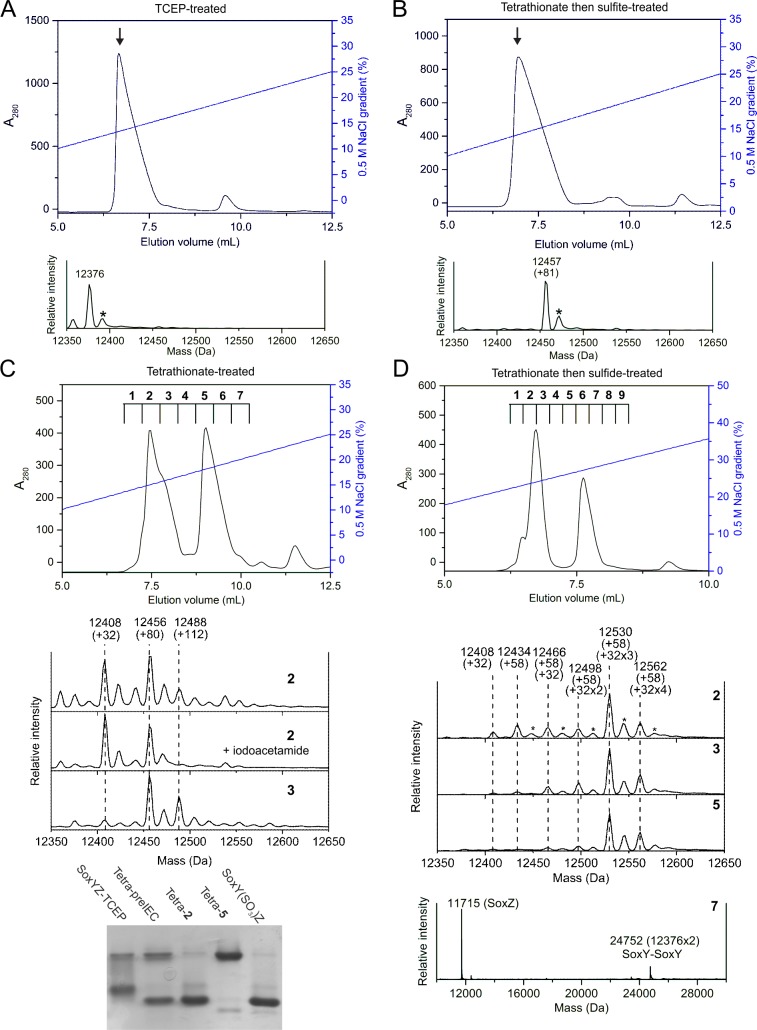
Generation of SoxYZ-conjugated candidate Sox pathway intermediates. SoxYZ was treated by the indicated protocols. The products were then analysed by ion exchange chromatography using a 1 mL MonoQ HR column (GE Healthcare) equilibrated in 30 mM Tris-HCl pH 8.0 (top of each panel). The fractions from the IEC separations that are indicated with arrows (A,B) or numbers (C,D) were analysed by ESI-MS and (for D only) by native PAGE (bottom of each panel). ESI-MS samples in (D) were treated with 20 mM iodoacetamide for one hour prior to analysis. The minor ESI-MS peaks marked with an asterisk (*) represent +15 Da adducts of the main peaks. Tetra-preIEC, Tetra-2, and Tetra-5 samples in (D) correspond to, respectively, the tetrathionate-treated SoxYZ before IEC and to the indicated fractions from the IEC separation.

SoxYZ with no modification of the substrate-bearing Cys residue was produced by purifying recombinant *P*. *pantotrophus* SoxYZ in the presence of the reducing agent TCEP. This TCEP-treated SoxYZ chromatographed as a single species during high resolution ion-exchange chromatography (IEC) (Fig 2A). ESI-MS confirmed that the SoxY Cys residue is unmodified (Fig 2A). As previously observed [6], an approximately +15 Da minor SoxY adduct is likely to be a species with either a methylated (+15 Da) or oxidised (+16 Da) methionine.

The SoxY(SO_3_)Z species was generated by reacting TCEP-treated SoxYZ successively with tetrathionate and then sulfite. Reaction with tetrathionate is expected to produce a S-thiosulfonate adduct of the carrier arm cysteine which will then undergo an exchange reaction with sulfite to give the cysteine-sulfonate adduct. IEC of the reaction product gave a single peak (Fig 2B) and ESI-MS of the peak fraction showed a SoxY modification of +80 Da, consistent with the presence of a sulfonate group (Fig 2B).

Preparation of the SoxY(SSO_3_)Z species has previously been attempted by reacting *P*. *pantotrophus* SoxYZ with tetrathionate. However, SoxY(SO_3_)Z was isolated in place of the intended product [6]. We found that incubating TCEP-treated SoxYZ with tetrathionate for shorter periods of time than used in the earlier study generated a mixture of products that could be partially separated by IEC (Fig 2C). ESI-MS of the earliest-eluting peak showed that the first part of the peak contained SoxY proteins with either a +80 Da modification, which is likely to be a sulfonate group, or a +32 Da modification, which potentially corresponds to a single sulfur atom in the form of a sulfane adduct (Fig 2C). To determine whether the +32 Da modification was indeed a sulfane group we treated the protein with iodoacetamide. Iodoacetamide reacts with free thiolate groups, such as cysteine-S-sulfane, to generate an amidocarboxymethyl derivative. No such derivative was seen by ESI-MS (Fig 2C), suggesting that the +32 Da species may instead be a cysteine where the thiol is oxidised to form a sulfinic acid (R-SO_2_^-^). The trailing shoulder of the first IEC peak showed an increase in a species at +112 Da which is likely to be the desired SoxY(SSO_3_)Z product (Fig 2C). No SoxY protein was detected by ESI-MS in the second IEC peak. However, native PAGE analysis showed that the SoxYZ species present in this peak had much retarded electrophoretic mobility relative to the SoxYZ protein in the first IEC peak or purified SoxY(SO_3_)Z (Fig 2C). This suggests that the protein in the second IEC peak corresponds to SoxYZ molecules that have been disulfide cross-linked together through their carrier arm cysteine residues as observed previously [11, 22, 23].

We attempted to produce sulfane and polysulfide derivatives of SoxYZ by subjecting the mixture of adducts produced through tetrathionate treatment of SoxYZ to thiol-disulfide exchange with a sulfide solution. The reaction products ran as two main peaks on IEC (Fig 2D). Eluted fractions from the IEC column were reacted with iodoacetamide to protect any acid-labile sulfane or polysulfide chains and then analysed by ESI-MS. Fractions across the first IEC peak revealed a series of related adducts comprising a single 58 Da amidocarboxymethyl group together with multiple 32 Da mass increments that are likely to correspond to increasing numbers of sulfur atoms (Fig 2D). The amidocarboxymethyl derivative of SoxY(SSS)Z is the most abundant of these conjugates but SoxY(S)Z, SoxY(SS)Z and SoxY(SSSS)Z derivatives as well as SoxYZ with no additional sulfur group were also detected. The second IEC peak contained disulfide-linked SoxY-SoxY (Fig 2D).

### The activity of different SoxYZ conjugates in a reconstituted Sox assay

Sox pathway activity can be reconstituted *in vitro* using the *P*. *pantotrophus* Sox pathway components SoxYZ, SoxAX, SoxB and SoxCD, together with thiosulfate as the electron donor and cytochrome *c* as the electron acceptor [14]. Any SoxYZ conjugate that is an authentic pathway intermediate would be expected to biochemically complement a reconstitution reaction lacking SoxYZ. We, therefore, attempted to reconstitute Sox activity using each of our *P*. *pantotrophus* SoxYZ preparations as the sole source of SoxYZ. Note that the small molecule reactants used in the preparation of the different SoxYZ conjugates had been removed during the sample work ups and were not, therefore, present in the reconstitution reactions.

**Fig 3:**
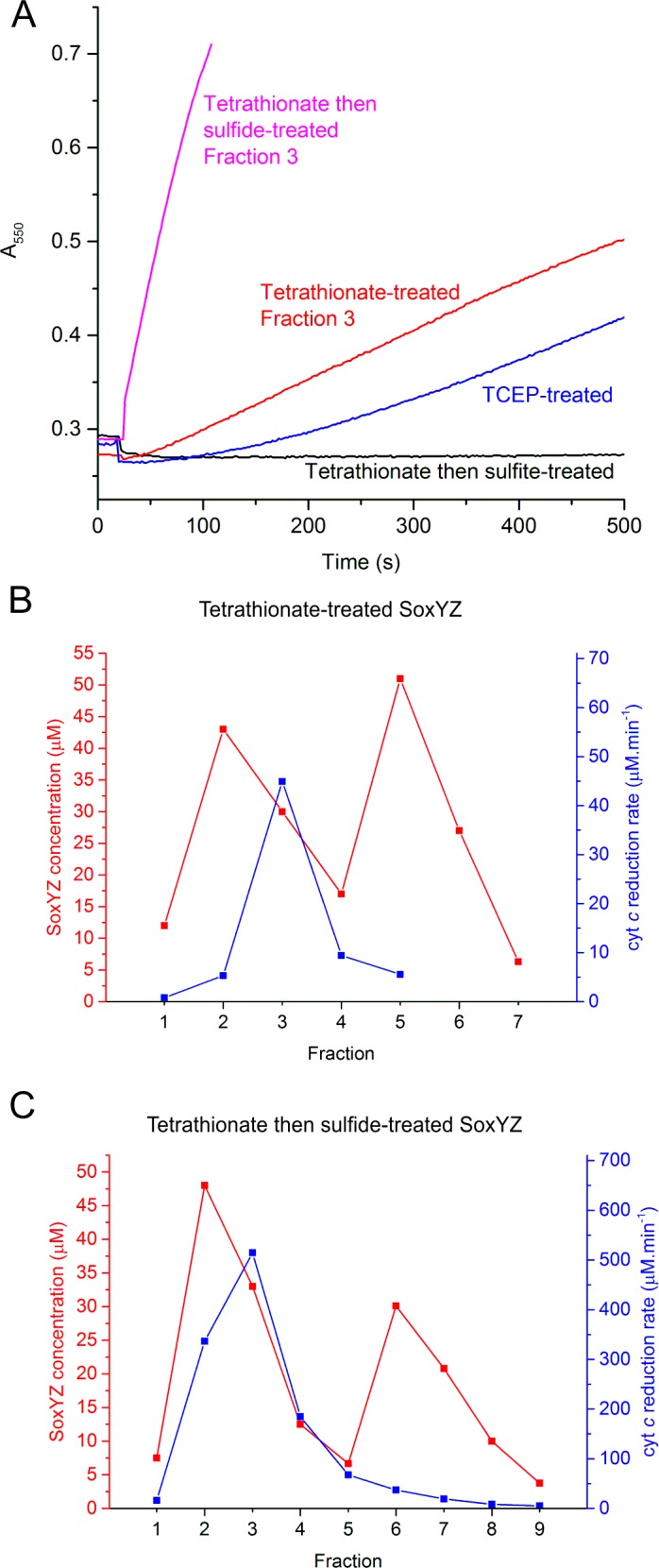
Thiosulfate oxidation activity of the Sox pathway reconstituted with different SoxYZ-conjugates. The SoxYZ conjugates were added to reactions mixtures containing SoxAX, SoxB, SoxCD, thiosulfate and cytochrome *c*. (A) Progression of Sox pathway activity for reconstitution reactions containing 0.1 μM of the indicated SoxYZ conjugates. Defined fractions are those characterized in panels (B) and (C). The reduction of cytochrome *c* was monitored at 550nm. IEC fractions of the (B) tetrathionate-treated and (C) tetrathionate then sulfide-treated SoxYZ preparations from Fig 2C and 2D, respectively, were assayed for activity. Fraction numbering is the same as in Fig 2C and 2D.

The underivatized SoxYZ produced by TCEP treatment was able to rescue the SoxYZ-depleted reconstituted Sox system, although only after a lag phase (Fig 3A). This shows that recombinant SoxYZ is functional but that it may require activation before it is utilised by the Sox pathway. By contrast, the Sox system complemented with SoxY(SO_3_)Z showed no thiosulfate-oxidising activity. This implies that SoxY(SO_3_)Z is not an intermediate in the Sox pathway (Fig 3A).

The other SoxYZ preparations did not contain homogeneously modified proteins. Consequently, we attempted to infer the functionality of the different species present by assessing how the relative proportions of the different species in different IEC fractions correlates to the ability of the fraction to biochemically complement the SoxYZ-deficient pathway.

For tetrathionate-treated SoxYZ, activity was only observed in the second half of the first IEC peak (Fig 3B) and, therefore, correlates with the presence of SoxY(SSO_3_)Z (Fig 2C). The active species in these fractions supported a higher pathway activity than TCEP-treated SoxYZ and the reaction trace did not exhibit a lag phase (Fig 3A). SoxY(SSO_3_)Z is therefore a credible Sox pathway intermediate.

For sulfide-treated SoxYZ, activity was maximum in the trailing part of the first IEC peak (Fig 3C). This suggests that the most active species are one or more of SoxY(S)Z, SoxY(SS)Z, SoxY(SSS)Z and SoxY(SSSS)Z (Fig 2D). The active species supported a higher pathway activity than either tetrathionate- or TCEP-treated SoxYZ and the reaction trace showed no lag phase (Fig 3A). Taken together these observations suggest that polysulfurated SoxYZ species are plausible Sox intermediates.

If a SoxYZ conjugate is an authentic Sox pathway intermediate then it should be possible for the pathway to use the conjugate as a substrate. This substrate turnover would be detected by our reconstitution assay if the reactions downstream of the intermediate result in the release of electrons. We investigated whether any of the putative pathway intermediates we had prepared were able to support turnover of the reconstituted Sox pathway in the absence of thiosulfate.

**Fig 4:**
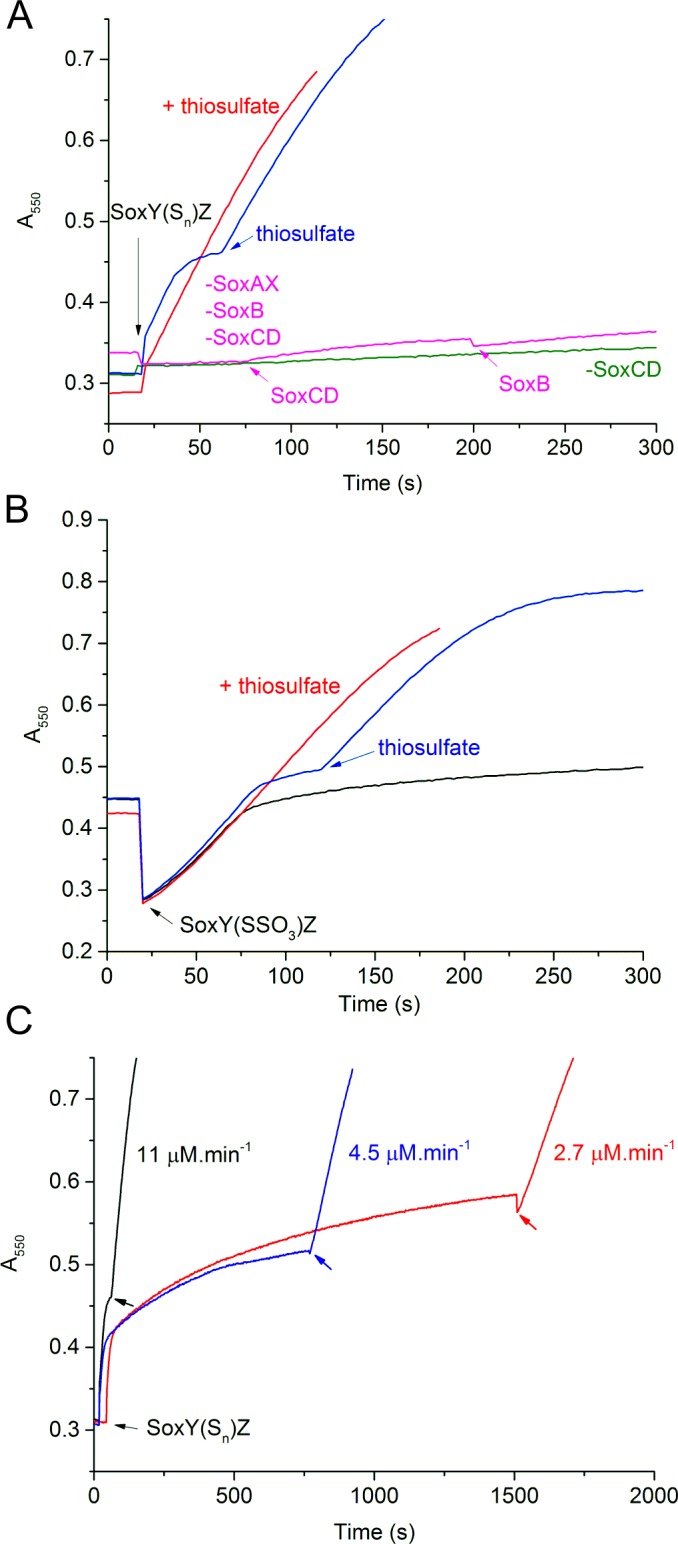
Thiosulfate-independent cytochrome *c* reduction. Reactions contained 0.1μM SoxB, SoxCD and SoxAX, unless indicated, and 35 μM cytochrome *c*. Any missing Sox components are indicated by a minus sign (e.g. `-SoxCD’). Reactions were initiated in panels (A) and (C) by the addition of 1 μM tetrathionate-then-sulfide-treated SoxYZ from fraction 3 of the IEX separation shown in Fig 3C (designated `SoxY(S_n_)Z’), or in panel (B) by the addition of 10 μM tetrathionate-treated SoxYZ from fraction 3 of the IEX separation shown in Fig 3B (designated `SoxY(SSO_3_)Z’). Where indicated, Sox components or thiosulfate were added to final concentrations of 0.1 μM or 2 mM, respectively. The progress of the reactions was assessed through monitoring the reduction of cytochrome *c* at 550nm.

Both tetrathionate and sulfide-treated SoxYZ preparations could be used as electron donors to the Sox system (Fig 4A and 4B) consistent with our earlier observations that both preparations contain SoxYZ conjugates that are able to function in the reconstitution reaction (Fig 3). Utilization of SoxY(SO_3_)Z as a substrate could not be assessed by this methodology because turnover of this adduct would not release electrons. Sulfide or tetrathionate-treated SoxYZ were initially rapidly oxidized but the rate of oxidation then quickly decreased (Fig 4A and 4B). This slowing of the reactions was due in part to exhaustion of substrate adducts because addition of thiosulfate was able to restore oxidation activity (Fig 4A and 4B). Nevertheless, for sulfide-treated SoxYZ, such thiosulfate additions recovered less activity if the initial reaction had been allowed to proceed further into the slow phase (Fig 4C). This suggests that non-functional forms of SoxYZ accumulate in the slow phase of the reaction. Oxidation of the sulfide-treated SoxYZ preparation required all three enzymes of the Sox pathway (Fig 4A and S1 Fig), including SoxAX which in the current pathway model would not be expected to be necessary for oxidation of any of the persulfurated SoxY species present (Fig 1A).

### Interaction of SoxYZ-conjugates with the active site metal ions of SoxB

The Sox pathway model predicts that SoxB uses both SoxY(SO_3_)Z and SoxY(SSO_3_)Z as substrates (Fig 1A). However, our reconstitution experiments suggest that SoxY(SSO_3_)Z is the only one of these two SoxY conjugates that is a Sox pathway intermediate (Fig 3). We were recently able to obtain thermodynamic evidence that the thiosulfonate group of the SoxY(SSO_3_)Z conjugate is capable of co-ordinating the two catalytic manganese ions in the SoxB active site [7], as required of a SoxB substrate. To obtain possible insight into why the sulfonate conjugate is not used as a pathway intermediate we have now undertaken an analogous thermodynamic analysis of complex formation between SoxB and SoxY(SO_3_)Z. These experiments were carried out with the SoxYZ and SoxB proteins of *Thermus thermophilus* and take advantage of the inability of the isolated SoxB enzyme to turn over its substrates [7].

**Fig 5:**
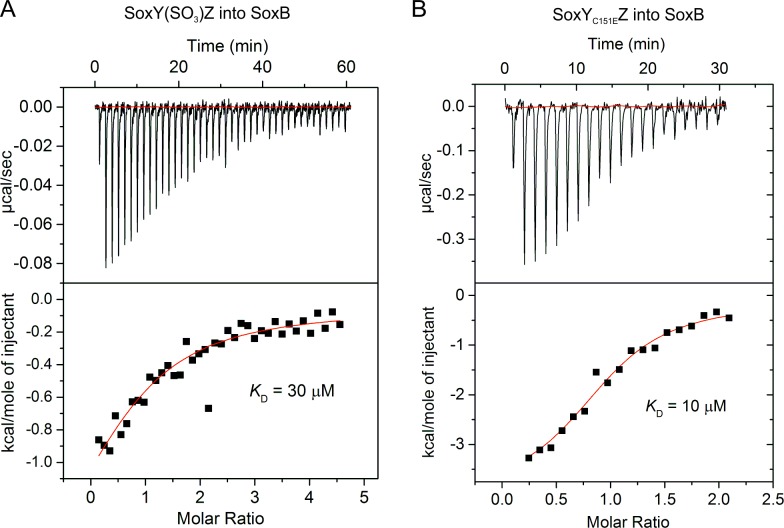
Thermodynamics of the interaction between *T*. *thermophilus* SoxYZ and SoxB measured by isothermal titration calorimetry. Representative titrations are shown but thermodynamic values were determined by fitting to duplicate titrations. (A) SoxY(SO_3_)Z was titrated into 50 μM SoxB. (B) SoxY_C151E_Z was titrated into 50 μM SoxB.

We found that the complex formed between SoxB and the inactive SoxY(SO_3_)Z conjugate is six-fold weaker than the complex formed between SoxB and the active SoxY(SSO_3_)Z conjugate (Table 1, Fig 5A). Thus, the active SoxYZ conjugate has a higher affinity for SoxB.

**Table 1:** Thermodynamic parameters for the interaction of *T*. *thermophilus* SoxB with different *T*. *thermophilus* SoxYZ conjugates.

Ligand	Side-chain structure	*K*_D_ (μM)	Δ*H* (kcal.mol^-1^)	Ref.
SoxY_C151S_Z	-CH_2_-OH	3	-3	[7]
SoxY(Am)Z	-CH_2_-S-**CH**_**2**_**-CONH**	12	-4	[7]
SoxY(SSO_3_)Z	-CH_2_-S-**S-SO**_**3**_^**-**^	5	+10	[7]
SoxY(Ac)Z	-CH_2_-S-**CH**_**2**_**-COO**^**-**^	0.7	+10	[7]
SoxY(SO_3_)Z	-CH_2_-S-**SO**_**3**_^**-**^	30	-2	This work
SoxY_C151E_Z	-CH_2_-CH_2_-**COO**^**-**^	10	-5	This work

The canonical Sox pathway model predicts that the product of the action of SoxB on SoxY(SO_3_)Z is unmodified SoxYZ (Fig 1A). We would expect an enzyme to have a higher affinity for the reaction substrate than it does for the reaction product. However, SoxY(SO_3_)Z forms a ten-fold weaker complex with SoxB than it does with SoxY_C151S_Z, which is an analogue of the predicted reaction product (Table 1).

We found that complex formation between SoxB and SoxY(SO_3_)Z is exothermic (Table 1, Fig 5A). We have previously observed that exothermic interactions between SoxB and SoxYZ are seen when SoxYZ is modified with a group which cannot bind to the dimanganese centre of SoxB, for example the cysteine-S-methylcarboxyamide group found in ‘SoxY(Am)Z’ or the unmodified side chain of SoxY_C151S_Z [7](Table 1). By contrast, endothermic interactions, correlate to the presence of groups that can co-ordinate the SoxB active site metal ions, such as those found in SoxY(SSO_3_)Z or its structural mimic carboxymethyl SoxYZ (‘SoxY(Ac)Z’)(Table 1). These correlations imply that the sulfonate group of SoxY(SO_3_)Z, in contrast to that of SoxY(SSO_3_)Z, does not co-ordinate the SoxB metal ions.

To test whether the differences in the interaction thermodynamics of SoxY(SO_3_)Z and SoxY(SSO_3_)Z with SoxB could be explained by the one atom difference in the length of the conjugated groups we examined the binding thermodynamics of SoxB with a pair of analogous species, SoxY(Ac)Z and SoxY_C151E_Z, that recapitulate the variation in side chain length. Whilst the longer conjugate SoxY(Ac)Z (side chain -CH_2_-S-CH_2_-CO_2_^-^) bound endothermically to SoxB, the shorter side chain variant SoxY_C151E_Z (side chain -CH_2_-CH_2_-CO_2_^-^) interacted exothermically and 10-fold more weakly with SoxB (Table 1, Fig 5B). That these binding differences mirror those seen between SoxY(SSO_3_)Z and SoxY(SO_3_)Z suggests that there is a minimal conjugate length for productive interaction with the SoxB active site.

## Discussion

The current model of the Sox pathway of thiosulfate oxidation predicts specific SoxYZ conjugates to be intermediates in the oxidation process (Fig 1). Here we have used biochemical complementation to test the validity of these SoxYZ conjugates as intermediates in the Sox pathway. Our results suggest that the current Sox pathway model is incorrect.

In the current Sox pathway model the S-sulfonate derivative of SoxYZ is predicted to be a substrate for the SoxB enzyme (Fig 1A). However, we found that SoxY(SO_3_)Z does not support Sox activity. We further observed that the sulfonate group of the conjugate is unable to co-ordinate the active site metal ions of the SoxB enzyme. Thus both reconstitution and binding experiments suggest that SoxY(SO_3_)Z is not an intermediate of the Sox pathway.

The underivatized form of SoxYZ is proposed to be part of the physiological Sox cycle (Fig 1A). However, we found that this form of SoxYZ only supported Sox pathway activity after a lag phase. This suggests that underivatized SoxYZ lies off the main Sox pathway and requires activation to enter the Sox pathway.

Other putative SoxYZ-linked intermediates could not be purified to homogeneity. Nevertheless, by correlating reconstitution activity to the species present in mixed populations of SoxYZ conjugates, we obtained presumptive evidence that SoxY(SSO_3_)Z and SoxYZ derivatives carrying chains of sulfur atoms are true Sox pathway intermediates. Indeed, the active fractions of these SoxYZ conjugates were also able to act as substrates for the Sox pathway (Fig 4).

The most parsimonious explanation for our observations is that the form of SoxYZ that carries pathway intermediates has at least one sulfane sulfur atom conjugated to the active cysteine residue. Viewed from this perspective underivatized SoxYZ and SoxY(SO_3_)Z lack the requisite sulfane atom and are unable (or are initially unable) to support Sox pathway activity. Conversely, the appropriate sulfane atom is present in the active SoxY(SSO_3_)Z and SoxY(S)_n_Z species. This model is supported by our biophysical analysis which suggests that a conjugated group without a sulfane sulfur atom is too short to reach the active site metal ions of SoxB. A possible chemical rationale for the requirement for a sulfane atom is that it is provides a better nucleophile than the unmodified cysteine. Alternatively, the larger and more hydrophobic sulfane-containing adducts may provide a handle for substrate recognition, as observed here and previously [7] for the interaction of SoxYZ conjugates with the active site of SoxB. A revised model for the Sox pathway based on these ideas is shown in Fig 1B. We note that neither the canonical, nor revised, Sox pathway models explain the observation that SoxAX is needed for the thiosulfate-independent oxidation of the sulfane-containing SoxYZ adducts (Fig 4A). The significance of this discrepancy is difficult to assess as unexplained dependencies between Sox enzymes are a general feature of the pathway [7, 18].

A previous study showed that SoxYZ purified directly from *P*. *pantotrophus* cells could be activated by treatment with sulfide, but that this activation did not occur if the SoxYZ preparation was first reacted with the reductant TCEP [23]. These observations were interpreted as showing that the active form of SoxYZ requires a specific SoxYZ conformation that is disfavored by TCEP treatment. However, the analysis presented here suggests an alternative explanation for these results. Natively-purified SoxYZ has a mixture of different species conjugated to the carrier arm cysteine of SoxY [5]. Addition of sulfide will convert these SoxYZ adducts to SoxY(S)_n_Z species as observed for the reaction of SoxY(SSO_3_)Z with sulfide in this study (Fig 2D). These SoxY(S)_n_Z species support high Sox pathway activity (Fig 3A and 3C). Treatment of the natively-purified SoxYZ with TCEP will remove any species conjugated to the carrier cysteine resulting in an underivatized form of SoxYZ that requires activation before it can participate in the Sox pathway (Fig 3A). With no leaving group available on the carrier arm cysteine the TCEP-treated SoxY protein cannot react with sulfide and therefore cannot be activated by sulfide treatment. Thus the chemical treatments undergone by SoxYZ in the earlier study differ in the extent to which they are able to add activating sulfur atoms to the carrier arm cysteine, and there is no need to invoke a conformational change in the SoxYZ protein to explain the observed effects on function.

In summary, biochemical complementation experiments show that SoxY(SO_3_)Z is not an intermediate during the oxidation of thiosulfate by the Sox pathway. We suggest a modified mechanistic model for the Sox pathway in which the intermediates are derivatives of SoxY(S)Z.

## Supporting information

S1 Fig Thiosulfate-independent cytochrome *c* reduction. Reactions contained 35 μM cytochrome *c*. Other reagents were added where indicated to the following final concentrations: 0.1μM SoxAX, 0.1μM SoxB, 0.1μM SoxCD, 2mM thiosulfate, and 1 μM tetrathionate-then-sulfide-treated SoxYZ (designated `SoxY(S_n_)Z’) from fraction 3 of the IEX separation shown in Fig 3C. The progress of the reactions was assessed by monitoring the cytochrome *c* reduction at a wavelength of 550nm.
(TIF)
