# Supplementary figures and images for: Intermediates in the Sox sulfur oxidation pathway are bound to a sulfane conjugate of the carrier protein SoxYZ
Source: PLoS One. 2017 Mar 3;12(3):e0173395. doi: 10.1371/journal.pone.0173395 (PMC5336275; doi:10.1371/journal.pone.0173395)

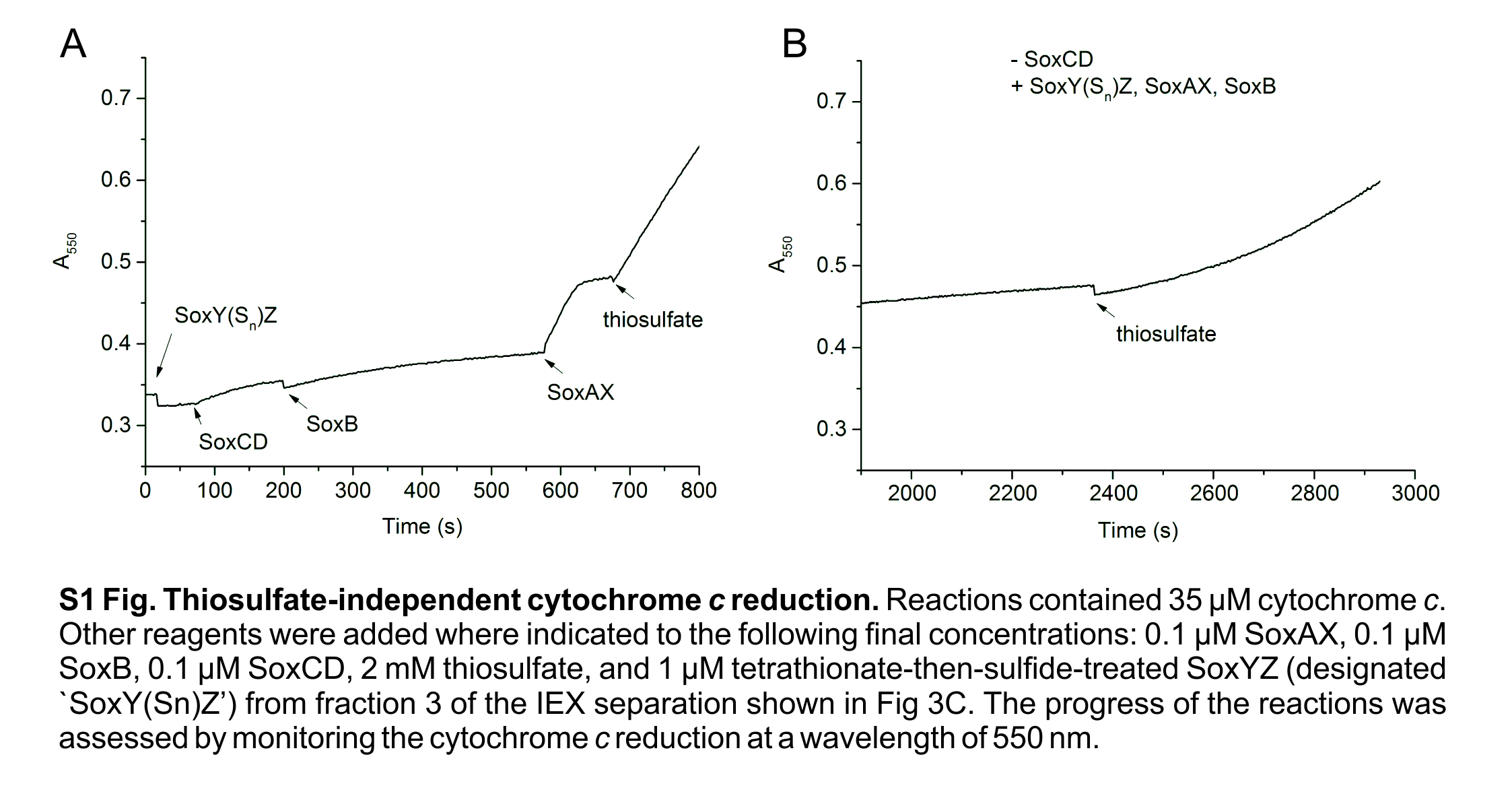

Supplement: S1 Fig — Reactions contained 35 μM cytochrome c. Other reagents were added where indicated to the following final concentrations: 0.1μM SoxAX, 0.1μM SoxB, 0.1μM SoxCD, 2mM thiosulfate, and 1 μM tetrathionate-then-sulfide-treated SoxYZ (designated `SoxY(Sn)Z’) from fraction 3 of the IEX separation shown in Fig 3C. The progress of the reactions was assessed by monitoring the cytochrome c reduction at a wavelength of 550nm. (TIF) [file pone.0173395.s001.tif]
